# Retracted: Application of Internet of Things Artificial Intelligence and Knowledge Innovation System in Table Tennis Teaching and Training

**DOI:** 10.1155/2022/9868093

**Published:** 2022-11-20

**Authors:** Applied Bionics and Biomechanics

*Applied Bionics and Biomechanics*has retracted the article titled “Application of Internet of Things Artificial Intelligence and Knowledge Innovation System in Table Tennis Teaching and Training” [1] due to concerns that the peer review process has been compromised.

Following an investigation conducted by the Hindawi Research Integrity team [2], significant concerns were identified with the peer reviewers assigned to this article; the investigation has concluded that the peer review process was compromised. We therefore can no longer trust the peer review process and the article is being retracted with the agreement of the Chief Editor.
